# Anti-CCL2 antibody combined with etoposide prolongs survival in a minimal residual disease mouse model of neuroblastoma

**DOI:** 10.1038/s41598-023-46968-2

**Published:** 2023-11-14

**Authors:** Danny Lascano, Michael J. Zobel, William G. Lee, Stephanie Y. Chen, Abigail Zamora, Grace E. Asuelime, So Yung Choi, Antonios Chronopoulos, Shahab Asgharzadeh, Araz Marachelian, Jinseok Park, Michael A. Sheard, Eugene S. Kim

**Affiliations:** 1 Division of Pediatric Surgery, Children’s Hospital Los Angeles, Los Angeles, CA USA; 2 Division of Hematology, Oncology and Blood and Marrow Transplantation, Keck School of Medicine, University of Southern California, Los Angeles, CA USA; 3 Department of Pediatrics, Keck School of Medicine, University of Southern California, Los Angeles, CA USA; 4 The Saban Research Institute, Children’s Hospital Los Angeles, Los Angeles, CA USA; 5 Department of Surgery, Cedars-Sinai Medical Center, 116 N. Robertson Blvd, Suite PACT 700, Los Angeles, CA 90048 USA; 6 Biostatistics and Bioinformatics Research Center, Cedars-Sinai Medical Center, Los Angeles, CA USA

**Keywords:** Paediatric cancer, Paediatric cancer

## Abstract

C–C motif chemokine ligand 2 (CCL2) is a monocyte chemoattractant that promotes metastatic disease and portends a poor prognosis in many cancers. To determine the potential of anti-CCL2 inhibition as a therapy for recurrent metastatic disease in neuroblastoma, a mouse model of minimal residual disease was utilized in which residual disease was treated with anti-CCL2 monoclonal antibody with etoposide. The effect of anti-CCL2 antibody on neuroblastoma cells was determined in vitro with cell proliferation, transwell migration, and 2-dimensional chemotaxis migration assays. The in vivo efficacy of anti-CCL2 antibody and etoposide against neuroblastoma was assessed following resection of primary tumors formed by two cell lines or a patient-derived xenograft (PDX) in immunodeficient NOD-scid gamma mice. In vitro, anti-CCL2 antibody did not affect cell proliferation but significantly inhibited neuroblastoma cell and monocyte migration towards an increasing CCL2 concentration gradient. Treatment of mice with anti-CCL2 antibody combined with etoposide significantly increased survival of mice after resection of primary tumors, compared to untreated mice.

## Introduction

Neuroblastoma is the most common extracranial solid tumor in children^1,2^. Approximately 80% of children with high-risk neuroblastoma will achieve remission following intensive, multimodal therapy, including surgery, radiation, ablative chemotherapy with autologous stem cell transplantation, and immunotherapy^2–8^. However, the 5-year event-free survival remains approximately 45%, with the majority of patients succumbing to refractory, recurrent disease^9,10^.

Despite improvements in survival of high-risk neuroblastoma patients after the introduction of anti-GD2 (disialoganglioside) immunotherapy, outcomes remain poor, and new therapies are needed to combat recurrent metastatic disease^9^. C–C motif chemokine ligand 2 (CCL2) or monocyte chemoattractant protein-1 (MCP-1) is known to attract monocytes to sites of metastasis and promote metastatic disease^10^. High levels of CCL2 are associated with a number of aggressive metastatic cancers, including breast, prostate, colorectal, and pancreatic cancers^11–14^. In vitro inhibition of CCL2 in these cancers has been found to inhibit a number of important metastatic mechanisms, such as reducing angiogenesis, decreasing tumor cell proliferation, ameliorating immunosuppression, reducing tumor resistance to chemotherapy, and reversing polarization of immune cells that would otherwise promote cancer progression^11–14^.

As the primary cause of death in children with high-risk neuroblastoma is the recurrence of widespread metastatic disease^9^, CCL2 is an attractive and rational target to counter tumor spread. However, the efficacy of anti-CCL2 antibody in preventing metastatic disease in neuroblastoma has not been studied. We utilized a metastatic model of minimal residual disease in immunodeficient NOD-scid gamma (NSG) mice that simulates the clinical setting in which metastatic disease follows surgical resection^15^. In this study, we demonstrate that anti-CCL2 antibody suppresses in vitro monocyte and neuroblastoma migration to CCL2, and when combined with chemotherapy, improves survival in our tumor resection mouse model of neuroblastoma.

## Results

### Increased CCL2 expression in patients with neuroblastoma is associated with death and progression of disease

**Figure 1:**
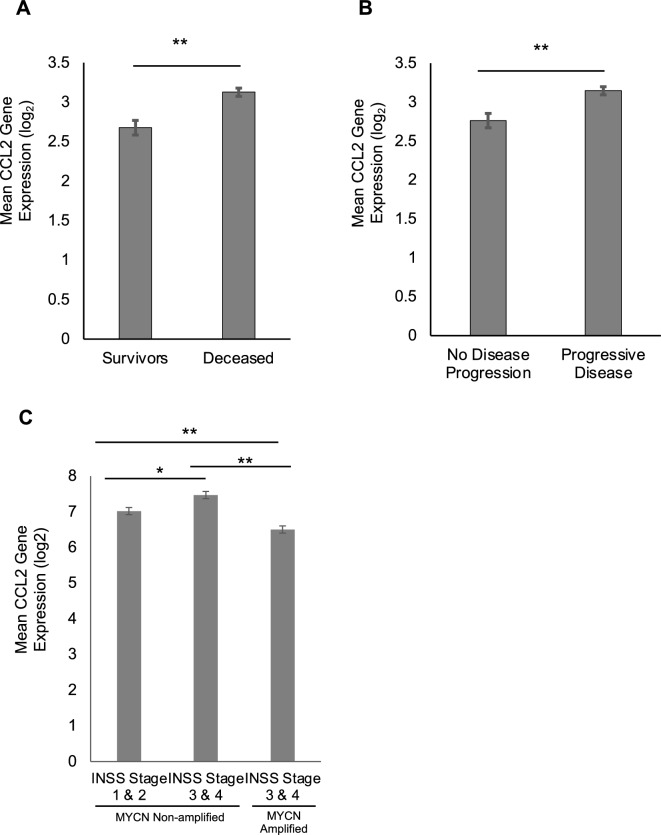
Correlation of CCL2 mRNA expression from neuroblastoma patients with survival and progressive disease. (**A)** Elevated mean CCL2 mRNA expression level is associated with increased mortality in neuroblastoma patients (Cangelosi et al. dataset)^16^. (**B)** Elevated CCL2 mRNA expression level is associated with progressive disease in neuroblastoma patients (Cangelosi et al. dataset)^16^. (**C)** Elevated CCL2 mRNA expression is associated with a higher INSS stage III/IV in non-MYCN amplified neuroblastoma patients, but not in MYCN amplified neuroblastoma patients^17,18^. Student’s t-test and one-way ANOVA of log-transformed data was performed; errors bars represent mean ± SEM; *p < 0.05, **p < 0.0001.

In order to assess the prognostic relevance of CCL2 gene expression in patients with neuroblastoma, RNA expression profiles, tumor biological characteristics, and clinical outcomes were obtained from available datasets previously analyzed by Cangelosi et al. and Asgharzadeh et al.^16–18^. Analysis of the database previously analyzed by Cangelosi et al. demonstrated elevated CCL2 RNA expression in patients that were deceased, compared to those still alive (Fig. 1A), as well as in patients with progressive disease defined as progression, relapse, or cancer-specific death (Fig. 1B). Analysis of the database previously used by Asgharzadeh et al.^17,18^ showed that elevated CCL2 RNA expression level correlated with advanced disease stage (i.e. stage III or IV) per the International Neuroblastoma Staging System Committee (INSS) in MYCN non-amplified tumors (Fig. 1C). Interestingly, CCL2 RNA expression level was significantly lower in MYCN amplified tumors compared to their MYCN non-amplified advanced disease stage counterparts (Fig. 1C). Survival analysis stratified by CCL2 RNA expression level showed no significant difference in event-free survival and overall survival (Supplementary Fig. S1). Based on these neuroblastoma patient datasets, increased CCL2 expression is observed in MYCN non-amplified tumors and is associated with advanced disease, disease progression, and deceased status, but showed no difference in survival.

### CCR2 is expressed on monocytes from neuroblastoma patients and neuroblastoma tumor cells

**Figure 2:**
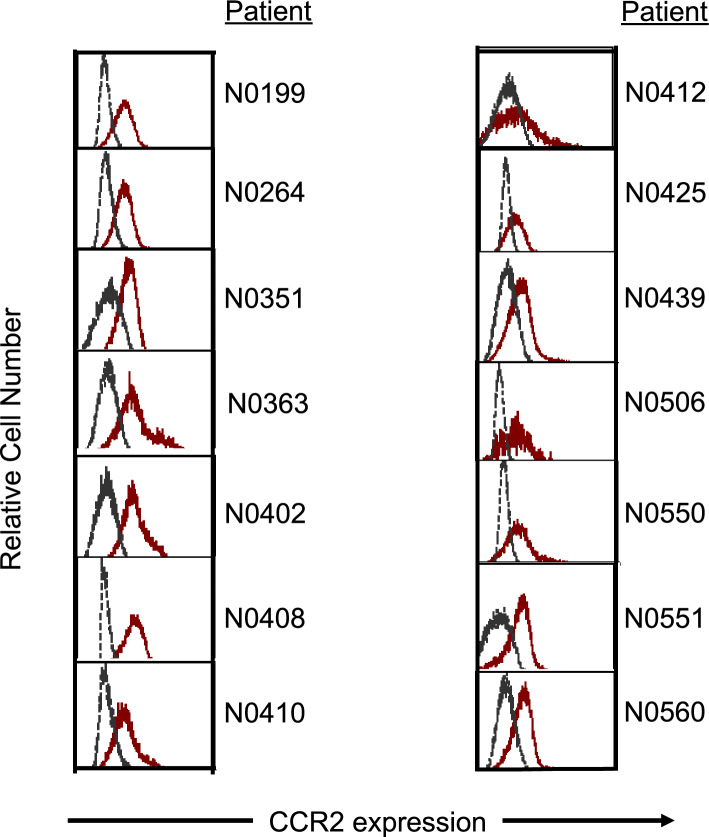
Prior to treatment, flow cytometry was performed on peripheral blood mononuclear cells obtained from 14 evaluable children with high-risk neuroblastoma. The presence of CCR2 was found on classical monocytes (red curve; CD45 + CD14 + CD16-) (From Marachelian et al. NANT-202211-04).

Flow cytometry was performed to measure surface expression of CCR2 (CD192), the native receptor for CCL2, on peripheral blood mononuclear cells obtained from 14 patients with neuroblastoma just prior to the initiation of systemic treatment (clinical trial NANT 2011-04, NCT01711554, provided by Dr. Araz Marachelian). Classical monocytes (CD45 + CD14 + CD16-) from 14 of 14 patients expressed a detectable level of CCR2 (Fig. 2). CCR2 was also found to be expressed in all 4 neuroblastoma cell lines examined, as well as a patient-derived xenograft tumor cells (Supplementary Fig. S2).

### CCL2 is predominantly released from monocytes with a minor contribution from neuroblastoma cells

**Figure 3:**
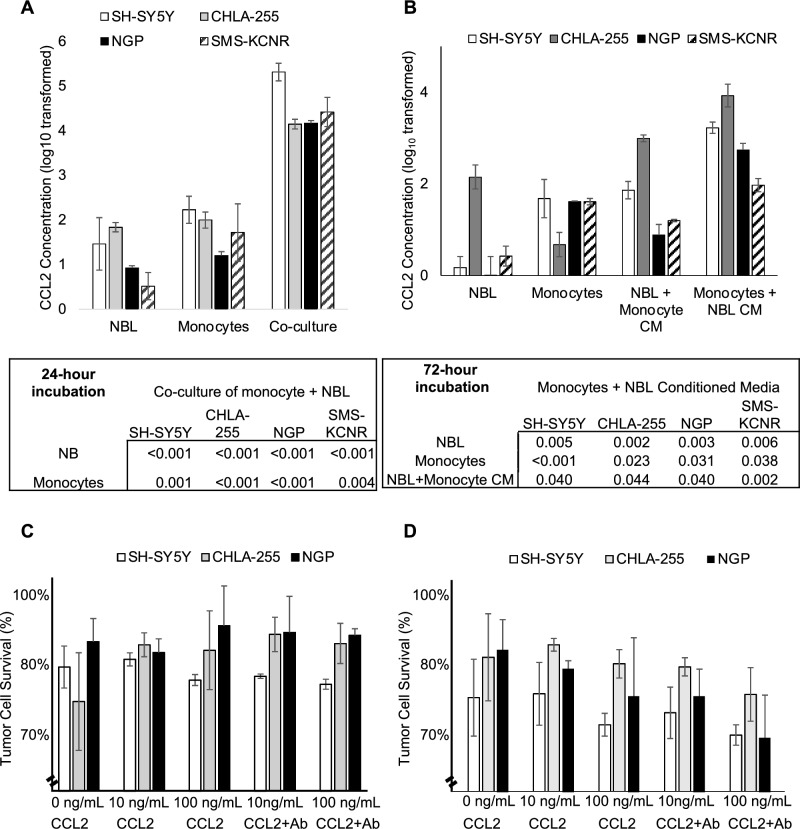
CCL2 expression from neuroblastoma cells and monocytes. (**A**) Utilizing four neuroblastoma cell lines (SH-SY5Y, CHLA-255, NGP, SMS-KCNR), neuroblastoma cells were co-cultured with monocytes, and CCL2 protein expression was measured by ELISA. Co-culture of neuroblastoma cells and monocytes leads to higher CCL2 expression compared to either alone. (**B)** To determine the primary source of CCL2 protein expression, monocytes were co-cultured with neuroblastoma supernatant and neuroblastoma cells were co-cultured with monocyte supernatant. Monocytes appear to contribute more CCL2 expression than neuroblastoma cells. Experiments were performed in quadruplicate for each cell line. Student’s t-test of log 10 transformed data was performed; errors bars represent mean ± SEM. The effect of CCL2 and anti-CCL2 antibody on neuroblastoma cells. (**C**) Cytotoxicity assays were performed on three neuroblastoma cell lines (SH-SY5Y-Fluc, CHLA-255-Fluc, NGP-Fluc) and incubated for 24 h and 48 h (**D)** in varying concentrations of recombinant CCL2, with and without anti-CCL2 antibody. The addition of CCL2, with and without anti-CCL2 antibody, did not affect neuroblastoma cell survival (*p* > 0.5 for all). ANOVA was performed; bar graphs and errors bars represent mean ± SEM.

Supernatants derived from 1:1 co-culture of any of four neuroblastoma cell lines (SH-SY5Y, CHLA-255, NGP, SMS-KCNR) with MACS-purified monocytes contained significantly higher levels of CCL2 protein than supernatant isolated from culture of either monocytes or neuroblastoma cells alone (Fig. 3A). To determine the primary source of CCL2, monocytes were cultured in neuroblastoma cell line supernatant and found to have a higher concentration of CCL2 protein compared to neuroblastoma cells cultured in monocyte supernatant (Fig. 3B). Moreover, neuroblastoma cells cultured in monocyte supernatant produced more CCL2 protein than neuroblastoma cells cultured without monocyte supernatant, although concentration levels were approximately a log lower than from monocytes. These results indicate that monocytes are the primary source of CCL2 protein produced during co-culture with neuroblastoma cells, with a lesser contribution from neuroblastoma cells.

### CCL2 has no effect on neuroblastoma cell proliferation in vitro

To determine the effect of CCL2 on neuroblastoma cell proliferation, four firefly-luciferase labeled neuroblastoma cell lines (SH-SY5Y-Fluc, CHLA-255-Fluc, NGP-Fluc, SMS-KCNR-Fluc) were cultured in increasing concentrations of CCL2. Luciferase cytotoxicity assay demonstrated no difference in tumor cell survival at varying concentrations of CCL2, after 24-h or 48-h incubation periods (Fig. 3C,D). Moreover, addition of antagonistic anti-CCL2 antibody to these assays did not affect the results demonstrating that the anti-CCL2 antibody does not have a cytotoxic effect on neuroblastoma tumor cells.

### In vitro, neuroblastoma cells and monocytes migrate towards increasing concentrations of CCL2, which is abrogated by anti-CCL2 antibody

**Figure 4:**
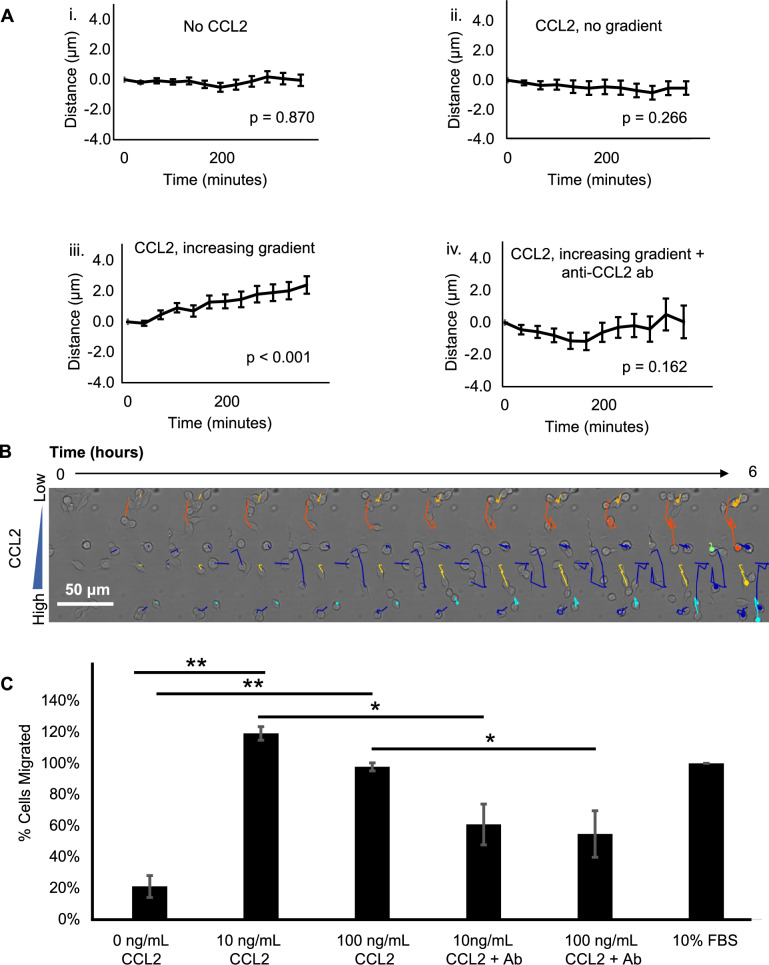
Neuroblastoma cell and human monocyte migration under varying concentration gradients of CCL2. (**A)** Utilizing time-lapse live cell microscopy, measured displacement of CHLA-255 cells towards varying CCL2 gradients was analyzed over time. Under conditions of no CCL2 (i) and CCL2 without a gradient (ii), there is no visualized neuroblastoma cell migration. Under the conditions of and increasing concentration gradient of CCL2 (iii), there is significant migration of neuroblastoma cells, which is abrogated by anti-CCL2 antibody (iv). (**B)** A representative image of measured neuroblastoma cell migration over time with the colored lines demonstrating the trajectory of the cell towards the increasing gradient of CCL2. Live cell microscopy data was performed in duplicate with over 100 cells measured per experiment. Linear regression analysis was performed for the live cell microscopy assay. (**C)** A monocyte migration assay utilizing a Boyden transwell migration assay with a CCL2 gradient was performed. Monocytes migrate toward increasing concentrations of CCL2, which is abolished by anti-CCL2 antibody. Percent migration was standardized to the positive control (10% FBS). Monocyte migration assay was performed in triplicate. Student’s t-test was used to analyze the monocyte migration assay data. Bar graphs and error bars represent mean ± SEM; *p < 0.05, **p < 0.01.

Two-dimensional time-lapse microscopy was performed to measure neuroblastoma cell movement across varying concentration gradients of CCL2 protein. Trajectory paths of over 100 randomly selected cells were analyzed to measure the mean kinetic effect. CHLA-255 neuroblastoma cells exhibited significant movement towards an increasing concentration gradient of CCL2, compared to tumor cells with no CCL2 and CCL2 with no concentration gradient. Moreover, the movement of the tumor cells in the increasing CCL2 concentration gradient is abolished with the addition of anti-CCL2 antibody (Fig. 4A). A schematic demonstrating a representative cell moving towards the CCL2 protein gradient is shown in Fig. 4B. Similar findings were demonstrated with a second neuroblastoma cell line, SK-N-SH (Supplementary Fig. S3). A monocyte migration assay was performed using Boyden transwell chambers with monocytes in the upper chamber and CCL2 in the lower chamber. A significantly greater number of monocytes migrated towards the CCL2 protein chamber compared to the chamber with no CCL2, and this effect was significantly inhibited by anti-CCL2 antibody (Fig. 4C). These results demonstrate that neuroblastoma cells and monocytes, which both express CCR2, migrate towards CCL2 protein, and that this effect is inhibited by anti-CCL2 antibody.

### The effect of carlumab on intra-tumoral tumor-associated macrophage (TAM) migration in vivo

To demonstrate the in vivo effect of anti-CCL2 antibody on the presence of TAMs within xenograft tumors, mice injected with CHLA-255-Fluc cells were treated with either anti-CCL2 antibody or control. CCL2 plasma protein expression was measured as described earlier and xenograft tumors underwent flow cytometry. Murine TAMs (mCD45 + Ly6G-mCD11b + F4/80 +) were quantified by flow cytometry (Supplementary Fig. S4B). The number of TAMs as a percentage of cells in xenografts trended higher in untreated mice versus treated mice (*p* = 0.07) (Supplementary Fig. S4C). When examining relative CCR2 expression level on TAMs, there was no difference between untreated and treated mice (Supplementary Fig. S4D). Plasma concentration of CCL2 increased markedly in all mice as tumor burden increased and was significantly greater in mice treated with anti-CCL2 antibody compared to untreated mice (Supplementary Fig. S4E). With the inhibition of CCL2 by the antibody treatment, this increased expression of CCL2 in the serum likely reflects a rebound positive feedback loop. Although the variability of TAMs present in the untreated control group limited significance, the trend observed in this data suggests that anti-CCL2 antibody may have the potential to decrease TAMs at sites of neuroblastoma.

### Anti-CCL2 antibody combined with chemotherapy increases survival in a murine tumor resection model of neuroblastoma

To determine the efficacy of CCL2 inhibition in vivo, a metastatic tumor resection model of minimal residual disease in mice was utilized. Following intrarenal injection of CHLA-255-Fluc or NGP-Fluc cells in mice and subsequent resection of the primary tumor, mice were treated with control (PBS), anti-CCL2 antibody alone, etoposide alone, or with the combination therapy of etoposide plus anti-CCL2 antibody.

**Figure 5:**
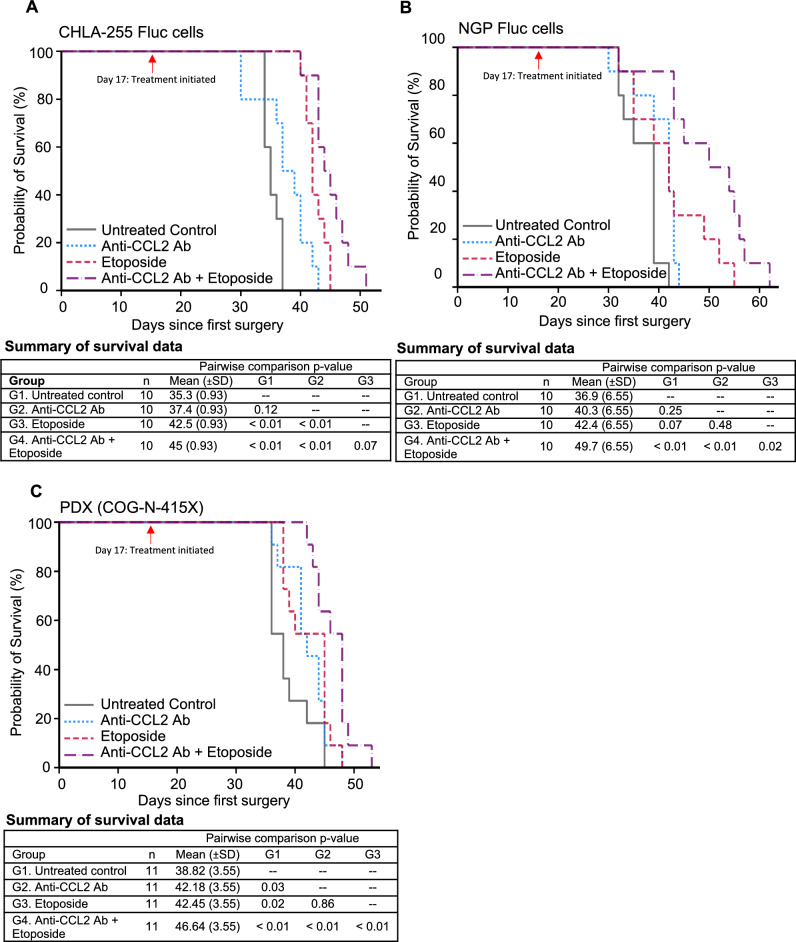
Combination therapy of anti-CCL2 antibody and etoposide improves survival in mice following surgical resection of primary tumors created from CHLA-255-Fluc (**A)**, NGP-Fluc (**B**), and PDX (COG-N-415X) (**C**). Kaplan–Meier survival plots shown here; differences in survival between groups were analyzed with linear regression.

In mice injected with CHLA-255-Fluc cells, mice treated with combination therapy had significantly increased survival compared to PBS mice (*p* < 0.01) and mice treated with anti-CCL2 antibody alone (*p* < 0.01) and trended towards increased survival compared to the etoposide alone group (*p* = 0.07) (Fig. 5A). In mice injected with NGP-Fluc cells, combination treatment with anti-CCL2 antibody and etoposide significantly increased survival compared to all other treatment groups (PBS control, *p* < 0.01; anti-CCL2 antibody alone, *p* < 0.01; etoposide alone, *p* = 0.02) (Fig. 5B). Finally, in mice injected with the patient-derived xenograft cells COG-N-415x, the mice treated with the combination of anti-CCL2 antibody and etoposide had significantly improved survival compared to all other treatment groups (PBS control, *p* < 0.01; anti-CCL2 antibody alone, *p* < 0.01; etoposide alone, *p* < 0.01) (Fig. 5C).

**Figure 6:**
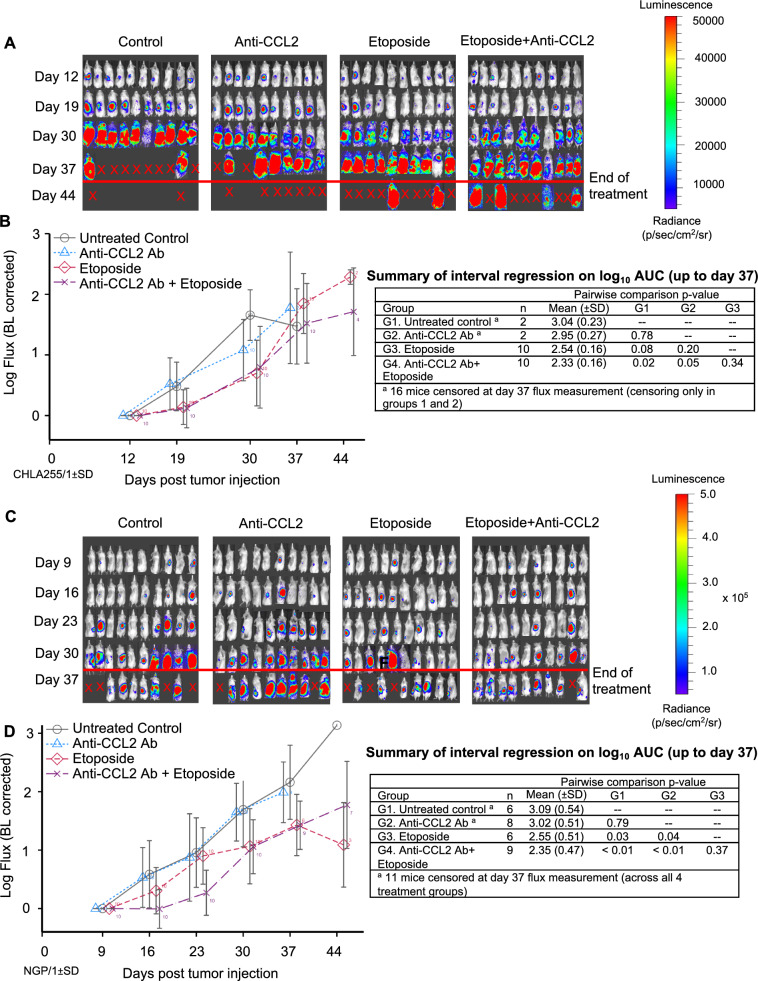
Combination treatment of etoposide with anti-CCL2 Ab decreases tumor burden in NOD-SCID gamma mice (NSG) injected with neuroblastoma (CHLA-255-Fluc and NGP-Fluc) in a minimal residual disease mouse model of neuroblastoma. (**A)** Following tumor resection of CHLA-255-Fluc orthotopic xenografts, mice were divided into four treatment groups (control, anti-CCL2 antibody alone, etoposide alone, and combination of etoposide and anti-CCL2 antibody) and underwent weekly bioluminescent imaging to measure tumor burden over time. (**B)** Combination therapy of anti-CCL2 Ab and etoposide led to significantly decreased tumor burden compared to untreated control. (**C)** Repeat experiment utilizing the NGP-Fluc neuroblastoma cell line. (**D)** Combination therapy of anti-CCL2 Ab and etoposide demonstrated decreased tumor burden compared to control. Interval regression was performed and line graphs with errors bars represent mean ± SD.

In addition to overall survival, we also examined the recurrence of metastatic tumor burden in mice. In CHLA-255-Fluc mice, combination therapy with anti-CCL2 antibody and etoposide led to a significant decrease in tumor burden compared to the PBS control group (*p* = 0.02) and the anti-CCL2 antibody alone group (*p* = 0.05), but not compared to the etoposide alone group (Fig. 6A,B). In NGP-Fluc mice, at earlier time points of treatment (up to days 16 and 23), the tumor burden (log-transformed tumor flux) of mice treated with anti-CCL2 antibody combined with etoposide was significantly inhibited over time compared to mice treated with PBS (*p* < 0.001), anti-CCL2 antibody alone (*p* < 0.001), or etoposide alone (*p* = 0.015) (Supplementary Table 2). This suggests that anti-CCL2 antibody and etoposide significantly suppresses recurrent metastatic disease during the early treatment period. By day 37, in the NGP-Fluc mice, combination therapy demonstrated a significant decrease in tumor burden compared to the PBS control group (*p* < 0.01) and the anti-CCL2 antibody alone group (*p* < 0.01), but not compared to the etoposide alone group (Fig. 6C,D).

## Discussion

After Yu and colleagues published their work demonstrating a marked improvement in outcomes in patients with high-risk neuroblastoma^19^, overall survival has increased with the addition of the immunotherapy dinutuximab. However, despite modest improvements in survival, approximately half of all children with high-risk neuroblastoma will eventually succumb to recurrent metastatic disease. Therefore, novel targets to inhibit and suppress metastatic disease are urgently needed.

CCL2 is a chemokine that attracts monocytes to the tumor microenvironment^10,11^ and facilitates the transformation of monocytes to tumor-associated macrophages (TAMs), which contribute to tumor cell growth, angiogenesis, and progression of metastatic disease in various cancers^10,20–22^. Upon analysis of gene expression profiles of neuroblastoma tumor samples, we found that increased CCL2 gene expression was associated with advanced disease, disease progression, and increased mortality. Interestingly, on subgroup analysis of MYCN non-amplified tumor samples, we found that CCL2 expression was higher in advanced stage tumors, while it exhibited decreased expression in MYCN-amplified advanced stage tumors. This may be due to epigenetic silencing in MYCN-amplified tumors which has been shown to decrease CCL2 expression in other MYCN-amplified tumors^23^. The binding of MYCN to E-box or initiator-like elements in the CCL2 promoter region interferes with transcription activation and subsequently represses CCL2 expression, independent of the STAT3 signaling pathway^24,25^. However, as MYCN amplification accounts for only 50% of all high-risk neuroblastoma cases, CCL2 may be a potential factor associated with increased risk in tumors that are not MYCN-amplified. Numerous studies have also demonstrated the role of CCL2 signaling in promoting advanced metastatic disease in breast^26^, prostate^27,28^, lung^29^, and hepatocellular cancers^30^. Preclinical studies have shown that neutralization of CCL2 decreases xenograft tumor progression^31^, inhibits metastasis, and prolongs survival in mouse models of breast cancer^11^ and prostate cancer^32^. Furthermore, CCL2 levels have been suggested to be a potential marker for disease surveillance following treatment of non-small cell lung cancer with anlotinib, a tyrosine receptor kinase inhibitor, which was found to suppress xenograft angiogenesis through CCL2 inhibition^33^.

Carlumab (CNTO 888, Janssen, Johnson & Johnson, CNTO888, Raritan, NJ) is a human monoclonal antibody against CCL2 that is currently being studied in clinical trials for its role as salvage therapy in advanced refractory solid tumors^34–36^. Early phase clinical trials by Sandhu et al. and Pienta et al. demonstrated the safety of carlumab in patients with treatment-resistant solid tumors with no dose-limiting toxicities reported^35,36^. These studies observed decreased circulating CCL2 levels with the combination of carlumab and other chemotherapeutic regimens^34–36^, suggesting targeted anti-tumor effects, but no prior studies have elucidated the optimal timing of carlumab initiation. Additionally, no studies have previously examined the role of CCL2 and carlumab in high-risk neuroblastoma.

To characterize the relationship between CCL2 and neuroblastoma, CCR2 expression, the primary receptor for CCL2, was confirmed in all neuroblastoma cell lines tested, as well as a PDX. Moreover, ubiquitous expression of CCR2 was identified on monocytes acquired from human patients with neuroblastoma. With regards to the expression of CCL2 itself, when neuroblastoma cells and monocytes were co-cultured, the expression of CCL2 was markedly higher than the expression of CCL2 from either cell population alone. Through subsequent studies, the primary source of CCL2 expression was from monocytes, but neuroblastoma cells, interestingly, also produce CCL2. This is also supported by an increased gene and protein expression of the CCL2 in recurrent/metastatic tumors, compared to the primary tumors (Supplementary Fig. S5). This suggests a model by which neuroblastoma cells that interact with monocytes produce increasing levels of CCL2, which in turn may attract more monocytes and neuroblastoma cells to sites of metastasis (Supplementary Fig. S6). This model is further supported by findings from a novel cell migration assay using time-lapsed microscopy. Neuroblastoma cells were found to have the greatest migration towards an increasing extracellular concentration gradient of CCL2, compared to experimental conditions which had no CCL2 or CCL2 without a concentration gradient. Monocytes were also found to migrate towards CCL2 in an in vitro transwell experiment. Thus, while CCL2 blockade may not exert a cytotoxic effect on tumor cells, anti-CCL2 agents may abrogate tumor cell migration and block the cycle of tumor cell recruitment to sites of metastasis—allowing for cytotoxic therapies to obtain control of the tumor cell population.

Finally, the neuroblastoma tumor resection mouse model of minimal residual disease was utilized to study the efficacy of anti-CCL2 antibody on survival. The most significant finding of this study was the increase in survival in mice treated with both anti-CCL2 antibody and the chemotherapy, etoposide. The in vivo survival benefit was reproducible using established neuroblastoma cell lines and a PDX model. The survival benefit was not observed in mice treated with etoposide alone, demonstrating the benefit of combining anti-CCL2 antibody with standard cytotoxic therapy. As neuroblastoma is a complex heterogeneous tumor, monotherapy is not recommended due to the elevated risk for treatment failure and drug resistance. The development of novel therapies aim to capitalize on alternative mechanisms of tumor cell survival, proliferation, and migration. Thus, preclinical evaluation of novel therapies for neuroblastoma often combines the use of a standard chemotherapy agent for neuroblastoma, such as etoposide, with the novel therapy of interest in order to evaluate its ability to augment/optimize existing cytotoxic agents^37–39^.

In this study, treatment with an anti-CCL2 antibody alone was not found to have a cytotoxic effect on neuroblastoma cells, which supports that the mechanism of action is via CCR2, the primary receptor for CCL2. CCR2 has been detected on T-cells, fibrocytes, natural killer cells, monocytes, and neuroblastoma cells^40–43^, but is expressed significantly more by monocytes than other immune cells. When monocytes migrate to tumor sites, they transform into TAMs, which promote angiogenesis, induce immunosuppression, and facilitate metastasis. As such, increased concentrations of TAMs at tumor sites have correlated with worse prognosis and local progression of disease, which may be due in part to a combination of promoting tumor growth and inhibiting anti-cancer mechanisms of the immune system^44,45^. Thus, targeting the CCR2-CCL2 pathway to inhibit metastatic disease in neuroblastoma is an attractive therapeutic target.

CCL2 contributes significantly to the pathogenesis of a number of cancers, including neuroblastoma, and other inflammatory conditions^46,47^. Inhibition of the CCR2-CCL2 pathway has been shown to reduce TAMs in metastatic animal models of breast, bladder, and colorectal cancer^11,48–50^. Qian and colleagues illustrated in a breast cancer model that CCL2 synthesized by metastatic tumor cells recruits CCR2-expressing monocytes, while inhibition of CCL2 markedly reduces the number of TAMs found in lung metastases^11^. Tu, et al. observed that shRNA silencing of CCL2 in a murine colon adenocarcinoma cell line attenuated liver metastasis, which correlated to decreased recruitment of TAMs to the site of metastasis^49^. Moreover, Chen, et al. demonstrated that suppressing CCL2 protein expression by inhibiting the upstream gene LNMAT1 decreased the presence of TAMs at metastatic sites for bladder cancer^50^.

In the current study, a delayed neuroblastoma tumor resection model in mice was utilized to study the effect of carlumab on cancer cell recruitment to tumor sites. Given the in vitro findings and the prolonged survival in mice treated with a combination of carlumab and etoposide, the inhibition of CCL2 by carlumab was hypothesized to suppress the recruitment of monocytes and neuroblastoma cells to tumor sites while etoposide would exert its cytotoxic effect on residual tumor cells. While curative therapy is the goal of novel cancer treatments, it is equally important to identify potential mechanisms that may prolong life. The findings from this study suggest that the early suppression of recurrent metastatic disease with the addition of carlumab may have contributed to the improved survival.

While this study supports the addition of anti-CCL2 antibody to etoposide in the treatment of metastatic neuroblastoma due to its survival benefit, the discrepancy between the clear survival benefit and the lack of sustained suppression of metastatic disease burden warrants further investigation and highlights the limitations of this experimental model. First, replication of in vitro results to an in vivo model can be subject to changes in environmental conditions. The tight control of the chemical and physical environment are clear advantages of in vitro research, but may fail to replicate in vivo environmental conditions. In vitro, the CCL2 concentration-dependent migration of neuroblastoma cells and monocytes was observed, which was abrogated with the addition of carlumab. However, in vivo, the effect of CCL2 inhibition on metastatic recruitment and TAM proliferation did not reach significance. Further investigation into in vivo extracellular CCL2 concentration gradients may add further clarity to the TME of neuroblastoma and its role in the progression of metastatic disease.

Another limitation of this model is the inherent immunodeficient nature in the NSG tumor model. Due to the NSG genetic background, mice lack their own T, B, and natural killer (NK) cells, and contain defects in dendritic cell development including monocytes. While NSG mice are commonly used in cancer models due to higher tumor engraftment rates and rates of metastasis, the discrepancy between TAM proliferation and survival benefit may be due to these defects in the monocyte and macrophage populations. The evaluation of carlumab in alternative immunocompetent models, such as humanized, transgenic, or syngeneic mouse models, could enhance the effect of CCL2 inhibition on monocyte migration and better elucidate the mechanism of cell recruitment to sites of metastasis.

Another proposed explanation behind the discrepancy between the survival benefit and lack of sustained disease suppression with combination therapy may be due to the acquisition of drug resistance. Although drug resistance to targeted therapies has been reported, as the anti-CCL2 antibody did not exert a cytotoxic effect, resistance to etoposide in this study may be more likely. Thus, the suppression of tumor growth at days 16 and 23 with combination therapy may have been due to etoposide-susceptible tumor cell death, followed by uncontrolled growth of etoposide-resistant tumor cells. This potential for rapid expansion of resistant cell populations highlights the importance of targeting multiple mechanisms in tumor cell survival and proliferation in neuroblastoma.

## Conclusion

In conclusion, inhibition of CCL2 suppresses CCL2-dependent cell migration of neuroblastoma cells and monocytes. In vivo, CCL2 inhibition combined with etoposide leads to increased survival in a surgical resection mouse model of minimal residual disease of neuroblastoma. Future study of CCL2 targeting in immunocompetent models of neuroblastoma may aid in elucidating the effect of CCL2 inhibition on tumor-associated macrophage proliferation and metastatic tumor burden.

## Materials and methods

### Database analysis of prognostic significance of CCL2 expression

To assess the prognostic relevance of CCL2 gene expression in patients with neuroblastoma, an analysis of RNA expression profiles of patients with neuroblastoma was performed. The objective was to evaluate for detectable differences in CCL2 gene expression in patients with unfavorable clinical outcomes. Two separate databases were analysed—one from a publicly available dataset previously reported by Cangelosi et al.^16^ and one from an unpublished dataset previously used by Asgharzadeh et al.^17,18^. Although Asgharzadeh et al. focused their analysis on metastatic tumors without MYCN gene amplification, the complete dataset which was used in this study also contained MYCN non-amplified and non-metastatic tumors^17,18^. These two databases were chosen for orthogonal validation as they represent the largest repositories of neuroblastoma tumor samples with reported CCL2 gene expression data. The first database reported by Cangelosi et al. compiled four datasets (RNA-seq498, Agilent709, Affymetrix413, Agilent262) and included the gene expression profiles for 786 untreated neuroblastoma primary tumor samples from patients across all risk groups after removing 834 unreliable or incomplete samples^16^. The second database used by Asgharzadeh et al. compiled single-institution gene expression data for 175 untreated neuroblastoma primary tumor samples with both MYCN amplified and MYCN non-amplified status^17,18^. Kaplan–Meier survival plots were used to estimate the survival function of CCL2 expression. Student’s t-test was performed on log2-transformed mean CCL2 expression between survivors and deceased, no disease progression and progressive disease, and International Neuroblastoma Staging System (INSS) stages 1–2 and stages 3–4. One-way analysis of variance (ANOVA) was performed on log2-transformed mean CCL2 expression between MYCN non-amplified early stage, MYCN non-amplified advanced stage, and MYCN-amplified tumors.

### Neuroblastoma cell lines and patient-derived xenograft

Human neuroblastoma cell lines SMS-KCNR, NGP, SH-SY5Y, and CHLA-255, and patient-derived xenograft (PDX) COG-N-415 × cells were derived from patients with progressive disease^51^. These cells were obtained from the Children’s Oncology Group Cell Culture and Xenograft Repository (http://www.cccells.org/).

SH-SY5Y-Fluc, SMS-KCNR-Fluc, NGP-Fluc, and CHLA-255-Fluc cells were created as previously described by stable transduction of the firefly luciferase (Fluc) gene using a lentivirus vector^17^. SH-SY5Y-Fluc and NGP-Fluc cells were gifted by Dr. Darrell Yamashiro (Columbia University, New York, NY, USA). CHLA-255-Fluc and SMS-KCNR-Fluc cells were gifted by Dr. Robert Seeger (Children’s Hospital Los Angeles, Los Angeles, CA, USA).

CHLA-255 was maintained in Iscove’s Modified Dulbecco’s Medium (IMDM). SMS-KCNR, NGP, and SH-SY5Y cells were maintained in RPMI-1640. Fetal bovine serum (FBS, 10%) was used to supplement both cell culture media, and this was supplemented with 2 mmol/L glutamine, 100 U/mL penicillin, and 100 mg/mL streptomycin at 37 °C in 5% CO_2_. PDX cells were cryopreserved at low passage (less than five) and expanded in vivo by subcutaneous injection into NSG mice.

Derived from a metastatic brain lesion from a patient, CHLA-255 cells overexpress c-MYC protein and lack MYCN amplification^17,51,52^. SH-SY5Ycells are non-MYCN-amplified and are a subclone of the SK-N-SH cell line, derived from a bone marrow biopsy of a patient^15^. SMS-KCNR cells were derived from a patient’s bone marrow and has MYCN amplification^53^. NGP cells were derived from a metastatic lung lesion and are MYCN-amplified^54^. COG-N-415 × patient derived xenograft have amplification of MYCN and mutation of ALK (F1174L).

SMS-KCNR, CHLA-255, SHSY-5Y, and COG-N-415X cells all express high levels of the disialoganglioside GD2 on their cell surface^55,56^. NGP has minimal GD2 expression on its cell surface^56^. Cell lines were routinely screened for mycoplasma contamination monthly using MycoAlert (Lonza, Cat# LT07-318, Allendale, NJ, USA). Neuroblastoma cell lines were authenticated by short tandem repeat multiplex assay using the AmpFLSTR Identifiler PCR Amplification Kit (Applied Biosystems, Cat# 4322288, Waltham, MA, USA).

### Reagents/assay preparation

Recombinant human CCL2 protein was obtained from R&D Systems (Minneapolis, MN, USA, Cat # 279-MC-050). Reagents used for in vivo experiments include etoposide (Toposar™, Teva Parenteral Medicine, Irvine, CA) and carlumab, a human IgG1κ monoclonal anti-CCL2 antibody donated by Janssen (Johnson & Johnson, CNTO888, Raritan, NJ)^34^. This antibody is specific to humans, and no cross-reactivity has been observed in other species^32^. To date, there are no preclinical or in-human studies that have evaluated the use of combination of anti-CCL antibody and chemotherapy agents in the treatment of neuroblastoma. Etoposide (VP-16) is a topoisomerase II inhibitor that is used extensively as a cytotoxic agent in standard chemotherapy regimens for neuroblastoma and has been previously established for concomitant therapy in the evaluation of novel therapies for neuroblastoma both in xenograft preclinical experiments and in-human trials^57–61^.

All cells used for in vitro assays were serum-starved for a minimum of 24 h or longer where indicated in either 0% FBS when in co-culture assays with monocytes or 2% FBS in migration and proliferation assays without monocytes. Serum starvation was used to synchronize the cell cycle of both monocytes and neuroblastoma cell lines used in these experiments to ensure bovine proteins did not interfere with detection of human CCL2.

### Human monocyte isolation, co-culture assays, and processing of conditioned media from culture supernatant

Leukocytes were obtained from healthy human donors at the Blood Center of our institution after approval from the Children’s Hospital Los Angeles (CHLA) Institutional Review Board. All experiments were performed in accordance with the relevant named guidelines and regulations. Informed consent was obtained from all subjects and/or their legal guardian(s). Leukocytes were collected as a by-product of the Trima Accel instrument (TerumoBCT, Lakewood, CO) used for platelet collection, from which peripheral blood mononuclear cells (PBMC) were obtained using Histopaque-1077 density centrifugal separation (Sigma-Aldrich, Cat# 1077, St. Louis, MO, USA). From these PBMCs, a negative selection EasySep Human Monocyte Isolation Kit (StemCell Technologies, Cat# 19359, Seattle, WA, USA) was used to enrich monocytes for subsequent assays as previously described^52,55^. The purity of monocytes was consistently greater than > 90% as determined by flow cytometry.

Immediately after enrichment, 1 × 10^6^ monocytes were co-cultured with neuroblastoma cells in a 1:1 ratio for 24 h, as per previously reported protocols^52,55^. This was repeated in quadruplicate for each cell line. Equal numbers of neuroblastoma cells and monocytes were also cultured separately in the same conditions and at the same concentration in serum-deprived media with no FBS. Supernatant was obtained from cultures as previously described^13,62^. This collected supernatant underwent centrifugation at 600 Relative Centrifugal Force (RCF) and subsequent sterile filtration using 0.45 µm polyethersulfone syringe filters to remove cellular debris and possible viral contaminants (VWR, Cat# 28145-505, Radnor, PA, USA).

Additional studies were performed to determine the primary source of CCL2 protein production. Four parallel culture conditions were established: (1) monocytes cultured in supernatant acquired from isolated neuroblastoma cell culture, (2) neuroblastoma cells cultured in supernatant acquired from isolated monocyte culture, (3) monocytes cultured alone, (4) neuroblastoma cells cultured alone. Supernatant from all four conditions were collected after 72 h and analyzed for CCL2 expression using enzyme-linked immunosorbent assay (ELISA).

### Cytotoxicity assay

1.5 × 10^4^ neuroblastoma cells (SH-SY5Y-Fluc, SMS-KCNR-Fluc, CHLA-255-Fluc, NGP-Fluc) were seeded per well in a 96-well plate for each treatment group. Neuroblastoma cells were either untreated or treated with recombinant CCL2 protein and incubated at 37 °C in 5% CO_2_ for 24 and 48 h. Neuroblastoma cell survival was assessed by luminescence 10 min after the addition of 50 ng of luciferin (Promega Corp., Cat# E160, Madison, WI). Cell survival for all groups was normalized to untreated cell survival which was maintained in 2% FBS alone. The cytotoxicity assays were performed in triplicate.

### Two-dimensional chemotaxis migration assay

To measure cell migration of neuroblastoma cells, a 2D chemotaxis assay was performed with neuroblastoma cells cultured on an Ibidi Collagen IV-coated µ-Slide (Ibidi, Cat# 80326, Gräfelfing, Germany). Serum-deprived neuroblastoma cells (18,000 cells in 6 µL of media) were seeded and allowed to adhere overnight. After confirming attachment, a protein gradient of CCL2 was generated by first filling 65 µL of serum-free media in the right and left reservoirs adjacent to the middle channel. 30 µL of CCL2 protein (4000 ng/mL) was applied to the bottom port of the left chamber and then the same volume was aspirated from the corresponding upper left port to diffuse the chemokine and create a chemotactic gradient. The comparison groups were CCL2 gradient (2000 ng/mL), CCL2 (2000 ng/mL) without gradient, CCL2 gradient (2000 ng/mL) with neutralizing anti-CCL2 antibody, and negative control with media only. Time-lapse imaging of the chemotactic movement of cells was conducted using an inverted Nikon Eclipse Ti2-E/B microscope housing a stage-top incubator to maintain appropriate temperature and CO_2_ levels (37 °C and 5% CO_2_) for the duration of the experiment. A 10 × objective was used in brightfield mode over six hours with a 30-min capture frequency. Over 100 cells were counted per condition in duplicate. After the time-lapse, the coordinates of individual cells were tracked using customized MATLAB codes.

### Monocyte migration assay

Monocyte migration/chemotaxis was measured utilizing a 96-well transwell migration assay utilizing a 5 µm pore size with migration into the lower chamber measured by fluorescence (Sigma-Aldrich, Cat# ECM512, St. Louis, MO, USA). Monocytes were used immediately after isolation and incubated in the upper chamber of a Boyden transwell migration with cells in the top compartment in serum-deprived media and the bottom compartment containing one of the following conditions: varying concentrations of CCL2 protein (10 mg/mL, 100 mg/mL) with no FBS, a positive control (10% of FBS), or a negative control (neither serum nor CCL2) separated by a semi-permeable membrane. 100,000 monocytes were added to each well into quadruplicate wells using RPMI 1640 media supplemented with 2 mmol/L glutamine, 100 U/mL penicillin, and 100 mg/mL streptomycin without phenol red (Thermo Fisher Scientific, Cat# 11835030, Waltham, MA USA). Cells were incubated at 37 °C and 5% CO_2,_ and after 12 h, cells were detached, lysed, and stained with CyQuant GR dye per manufacturer protocol. Fluorescence in the lower well was measured using a microplate reader with a 480/520 nM filter set per standard protocol.

### CCL2 protein expression studies

Serum CCL2 protein levels were measured in conditioned media from cultured supernatant or murine plasma using a human CCL2/MCP-1 Quantikine ELISA kit (R&D Systems, Cat# DCP00, Minneapolis, MN, USA). Briefly, for in vitro experiments, supernatant was collected from co-culture assays as previously described and was diluted with assay diluent media as per manufacture’s protocol and probed for CCL2 protein via ELISA. For the in vivo experiments, murine blood was collected and processed for plasma analysis both before (i.e. baseline) and after xenograft tumor implantation on a weekly basis. Plasma was collected in treated and untreated mice and cryopreserved at − 80 °C until batched analyzed at 12 weeks post-tumor implantation. Samples were thawed, diluted per manufacturer’s protocol, and probed for CCL2 protein via ELISA using a murine anti-CCL2 assay (Mouse CCL2/JE/MCP-1 DuoSet ELISA, Catalog #: DY479, R&D).

### In vivo* murine experiments and bioluminescent imaging*

NSG mice were bred in-house under pathogen-free conditions per institutional protocols. All animal experiments were performed in accordance with the relevant guidelines and regulations—and were approved by the Institutional Animal Care and Use Committee of CHLA (IACUC #363-20). This study is reported in accordance with the ARRIVE guidelines. Male and female, 6–8-week-old NSG mice were used for all in vivo experiments. CHLA-255-Fluc, NGP-Fluc, and COG-N-415X neuroblastoma cells were cultured, harvested, counted, and suspended at a concentration of 1 × 10^7^ neuroblastoma cells per mL in sterile PBS.

Xenograft experiments were performed using a previously described murine model of minimal residual disease^15,63^. Two separate xenograft experiments were performed. The first xenograft experiment evaluated the in vivo effect of anti-CCL2 antibody on the presence of tumor-associated macrophages (TAMs) at metastatic sites. This experiment deviated from the published protocol as follows. Briefly, under anesthesia (isoflurane aerosolized anesthesia and bupivacaine local anesthetic), the left kidney of the anesthetized mouse was exteriorized, and 1 × 10^6^ neuroblastoma cells were injected beneath the renal capsule^15,63,64^. The kidney was returned to the retroperitoneal space, the muscle closed with a single 4–0 Vicryl suture (Ethicon, Cincinnati, OH), and the skin closed with a skin clip. Neuroblastoma xenografts were allowed to grow, and starting on post-tumor cell injection day 24, mice were treated daily for 7 days with either anti-CCL2 antibody (2 mg/kg/dose/mouse) or control (PBS) via intraperitoneal injection. After 34 days, mice were sacrificed and xenograft tumors were processed (Supplementary Fig. S4A).

The second xenograft experiment evaluated the efficacy of anti-CCL2 antibody. This experiment followed the described murine model of minimal residual disease as published^15,63^. In this experiment, bioluminescent imaging of all mice was performed 12 days following the initial injection to facilitate matched treatment groups with 10 mice per group. The treatment groups included the following: (1) untreated control (PBS), (2) anti-CCL2 antibody (2 mg/kg/dose/mouse), (3) etoposide (8 mg/kg/dose/mouse), or (4) anti-CCL2 antibody (2 mg/kg/dose/mouse) + etoposide (8 mg/kg/does/mouse). All treatments began three days after tumor resection (post-tumor cell injection day 17) and continued twice weekly for five weeks via intraperitoneal injection. Total burden of local recurrent and metastatic disease for each mouse was monitored weekly for five weeks using bioluminescent imaging (Xenogen IVIS Lumina XR System, Caliper Life Sciences, Waltham, MA, USA) and quantified by calculating total flux (photons/second) for each mouse using Living Image software (Living Image 4.3.1, Caliper Life Sciences, Waltham, MA)^63^. Mice were monitored daily for survival and euthanized (CO2 euthanasia followed by cervical dislocation) when IACUC criteria were met, including lethargy, poor grooming, weight loss, and/or hind-limb paralysis. Treatment with etoposide, anti-CCL2 antibody (carlumab), or combination therapy (etoposide + carlumab) was well tolerated in all mice without any observed adverse effects.

This established model of minimal residual disease has been shown to consistently excise all of the primary tumor, followed by tumor growth in the liver and femur (bone marrow), which are common sites of neuroblastoma metastasis^15^. The primary investigator (E.S.K.) who performed all of the tumor resections is an attending pediatric surgeon with 23 years of experience with this mouse model. In this model, the entirety of the primary tumor is confined to the kidney which is completely resected (i.e. nephrectomy) at the time of primary tumor resection. Thus, all tumors that develop post-resection comprise recurrent or metastatic disease. This is indicated by the lack of bioluminescent signal post-resection, followed by the delayed presence of bioluminescent signal.

### Flow cytometry

At the time of sacrifice (CO2 euthanasia followed by cervical dislocation), the primary tumor with the injected kidney (if not previously removed), the contralateral kidney (as a control), and all organs containing gross metastatic disease were collected for histopathologic analysis. These organs included the liver and the bone marrow from the bilateral femurs. Upon pathologic confirmation of neuroblastoma cell infiltration, all tumor tissues were processed into single-cell suspensions. Bone marrow, primary tumors, and control kidneys were isolated into single-cell suspension using tumor cell isolation kits (Miltenyi Biotec Inc., Cat # 130-095-929, North Rhine-Westphalia, Germany). Livers were processed using 150 µL of collagenase I (100 CDU/mL; Sigma-Aldrich Cat# 0130, St. Louis, MO, USA), 150 µL of dispase II (32 mg/mL; Roche, Cat# 04942078001, Basel, Switzerland), and 2 µL of DNase I (2 mU/mg; Calbiochem, Cat # 260913, Darmstadt, Germany) on ice while undergoing mincing and remained in this mixture during mechanical dissociation for 60 min at 37 °C with further mechanical mincing using a gentleMACS Tissue Dissociator (Miltenyi Biotec Inc., North Rhine-Westphalia, Germany). Single-cell suspensions were counted by trypan blue exclusion.

To confirm neuroblastoma cells, single-cell suspensions of liver and bone marrow were diluted in freezing medium (20% FBS, 72% RPMI 1640, 8% DMSO), frozen slowly in a dedicated container at − 80 °C for one day, and transferred to a liquid nitrogen freezer for longer-term storage. Cells were then gently thawed and cellular debris removed by washing in FACS buffer (PBS + 0.5% bovine serum albumin or BSA + 1 mM ethylenediaminetetraacetic acid or EDTA) three times. Samples were resuspended and stained with 4′,6-diamidino-2-phenylindole (DAPI) nucleic acid viability dye to enable dead cell exclusion (Sigma-Aldrich, Cat # D9542). Neuroblastoma cells were identified by double positivity after staining with anti-GD2-BV605 antibody (clone 14.G2 α, BD Biosciences, Cat # 74071, Franklin Lakes, NJ, USA) and anti-CD56-BUV395 (clone NCAM16.2, BD Biosciences, Cat # 563554, Franklin Lakes, NJ, USA). Cell suspensions were filtered through a 35-micron nylon strainer before analysis by flow cytometry using a BD LSRII (BD Biosciences, San Jose, CA) and BD FACSDiva Software (Version 6.1.3, BD Biosciences, San Jose, CA).

To identify and quantify TAMs present in primary tumors, samples were analyzed by flow cytometry, without freezing. Single-cell suspensions were blocked with human TruStain FcX, an Fc-receptor blocker for 10 min (Biolegend, Cat # 422302, San Diego, CA, USA), stained with antibodies for 30 min, and washed three times in FACS buffer (PBS + 0.5% BSA + 1 mM EDTA). Antibodies used to identify TAMs (mCD11b, mCD45, F4/80, Ly6C, and Ly6G) and neuroblastoma cells are listed in Supplementary Table 1. After the final wash, samples were filtered through a 35-micron nylon strainer, resuspended, and stained with DAPI for dead cell exclusion (Sigma-Aldrich, Cat # D9542).

### Statistical analysis

Data from in vitro assays were compared using Student’s t-tests. Area under the curve (AUC) for the tumor group was calculated from tumor flux measurements adjusted to baseline observations. AUC was log10 transformed to normalize data. AUC was calculated using the last flux measurements obtained before euthanasia. AUC was censored if/when flux measurements were missing, in cases when mice were euthanized before reaching the time point for bioluminescent reading. Survival time was defined as the length of time between the day of tumor cell injection and the day when mice were sacrificed due to disease burden. Interval regression was used to analyze the tumor growth model. Linear regression examined treatment group differences on log10-transformed AUC and survival time. Analysis of survival time was repeated on log10-transformed survival time. To describe the rate of change in tumor growth in the earlier intervals (up to day 23), a linear mixed-effects model was fitted for log-transformed tumor flux data in mice injected with NGP-Fluc cells which adjusted for treatment group, days from tumor cell injection, and interaction between treatment group and time. Analyses presented here were performed using Stata 17 (StataCorp LLC, College Station, TX) or R version 4.2.2 (R Core Team, Vienna, Austria), and unless otherwise noted, *p*-values refer to two-sided tests. A *p*-value < 0.05 was considered significant.

### Supplementary Information

Supplementary Figure S1.

Supplementary Figure S2.

Supplementary Figure S3.

Supplementary Figure S4.

Supplementary Figure S5.

Supplementary Figure S6.

Supplementary Information 7.

## Data Availability

The datasets generated during and/or analyzed during the current study are available from the corresponding author upon reasonable request.
