# Supplementary figures and images for: Anti-CCL2 antibody combined with etoposide prolongs survival in a minimal residual disease mouse model of neuroblastoma
Source: Sci Rep. 2023 Nov 14;13:19915. doi: 10.1038/s41598-023-46968-2 (PMC10645976; doi:10.1038/s41598-023-46968-2)

Supplementary Figure S1

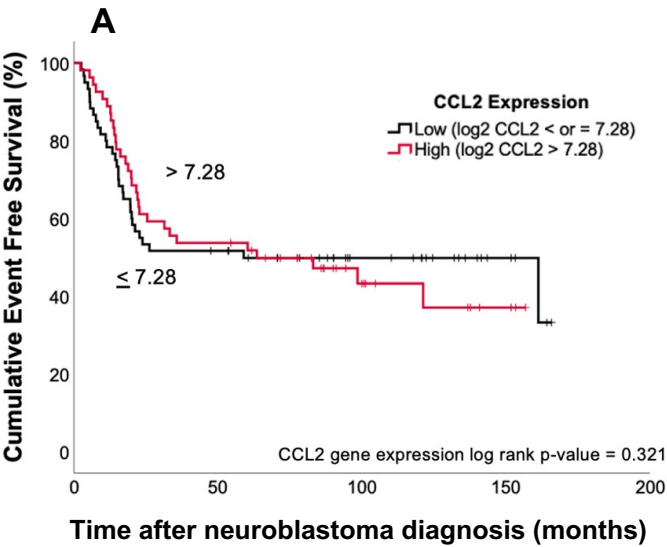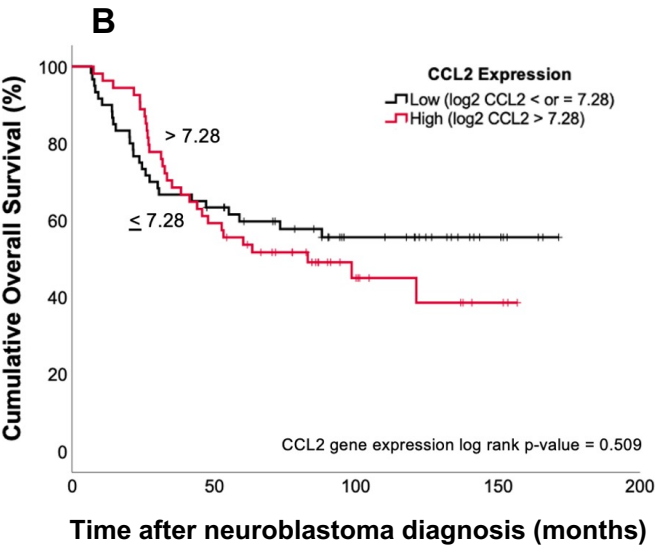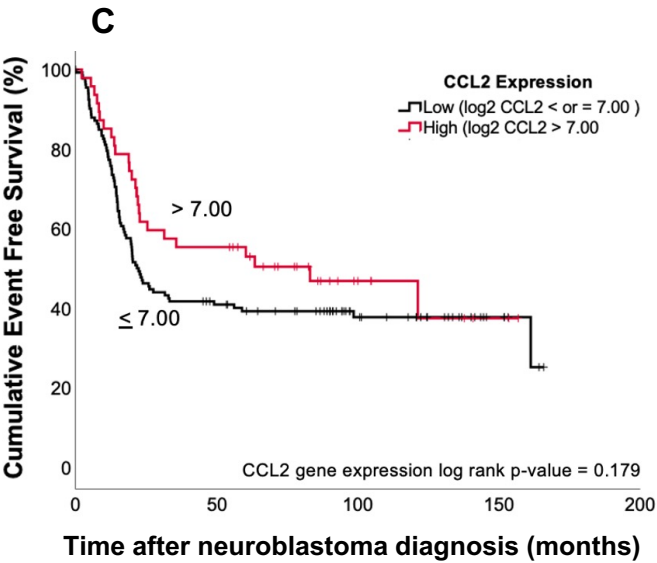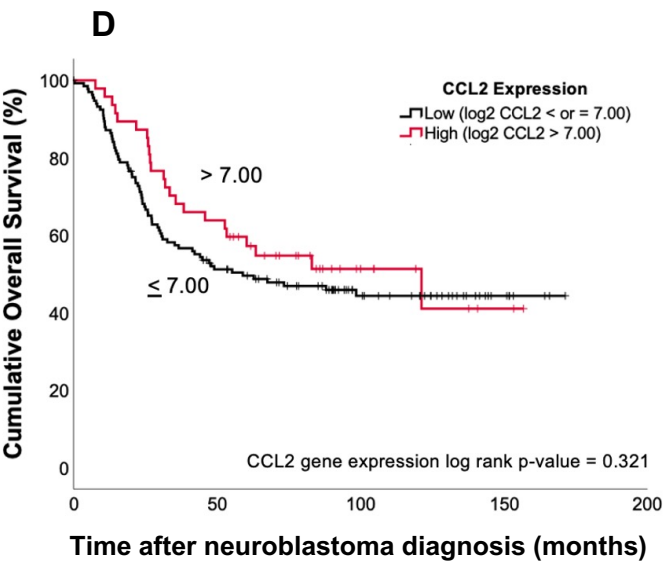

Supplement: Supplementary file 1 — Supplementary Figure S1. [file 41598_2023_46968_MOESM1_ESM.pdf]

Supplementary Figure S2

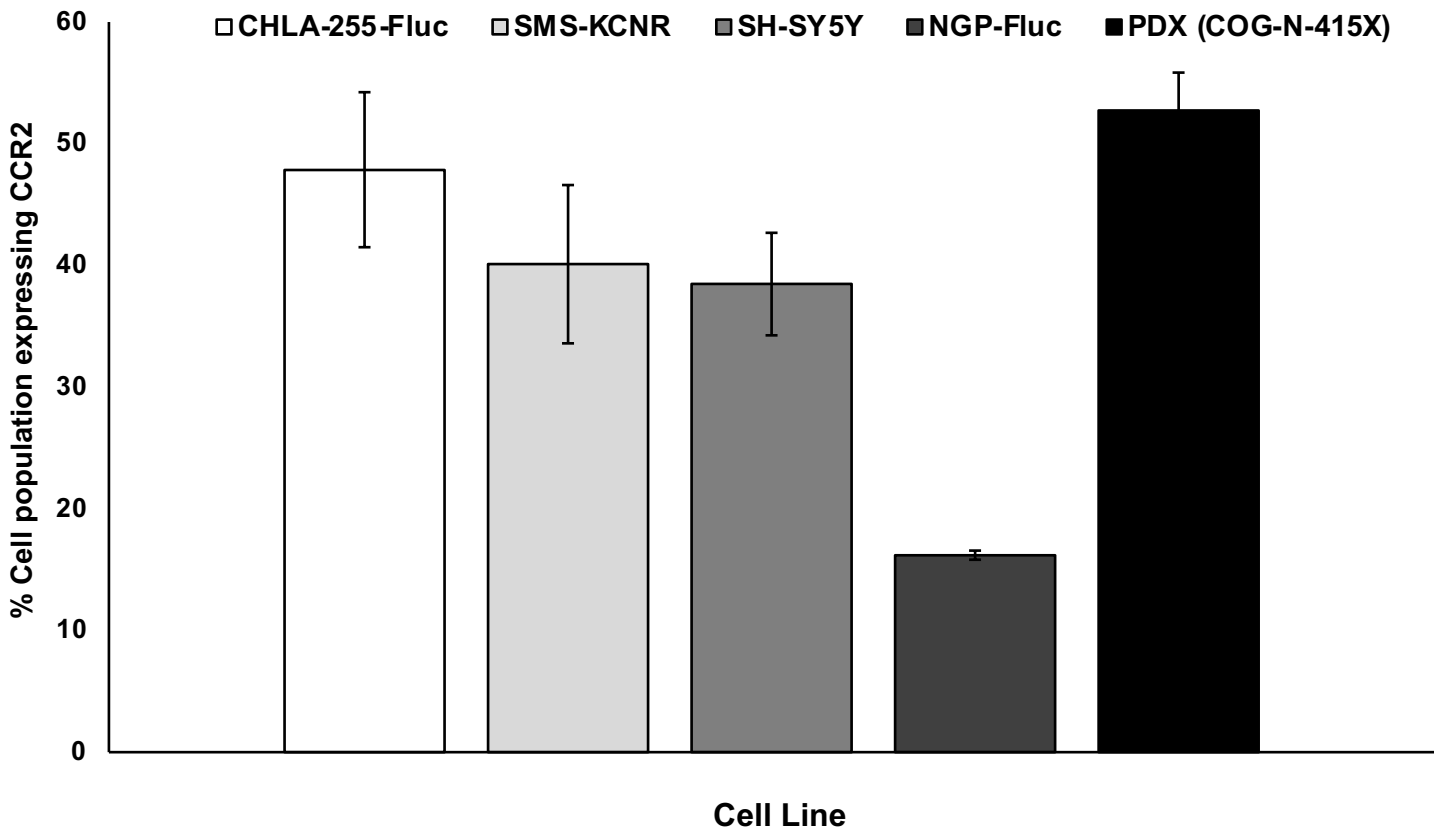

Supplement: Supplementary file 2 — Supplementary Figure S2. [file 41598_2023_46968_MOESM2_ESM.pdf]

Supplementary Figure S3

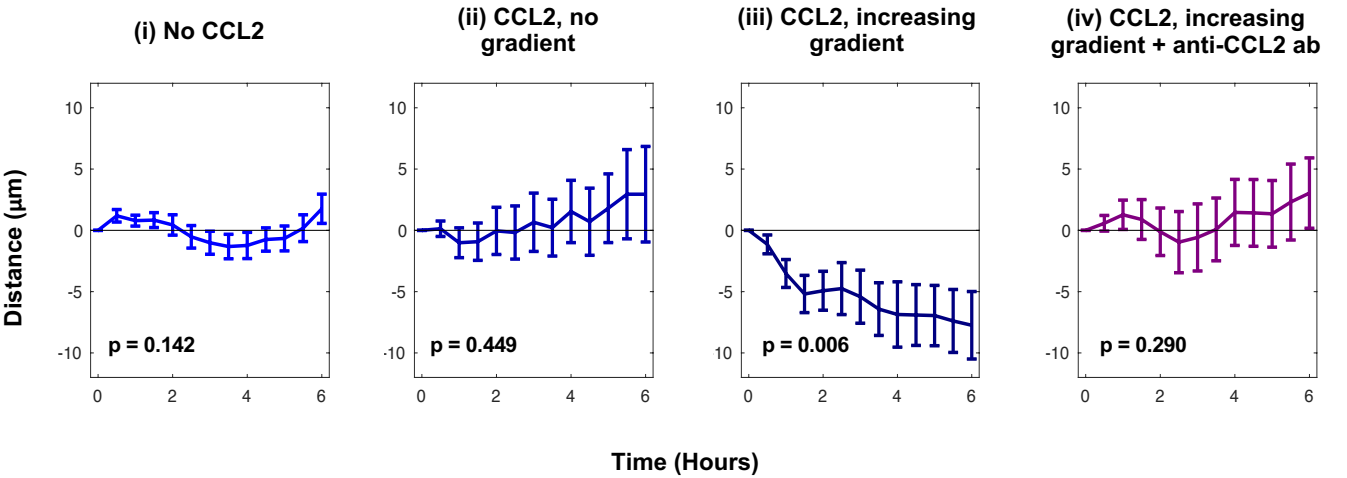

Supplement: Supplementary file 3 — Supplementary Figure S3. [file 41598_2023_46968_MOESM3_ESM.pdf]

Supplementary Figure S4

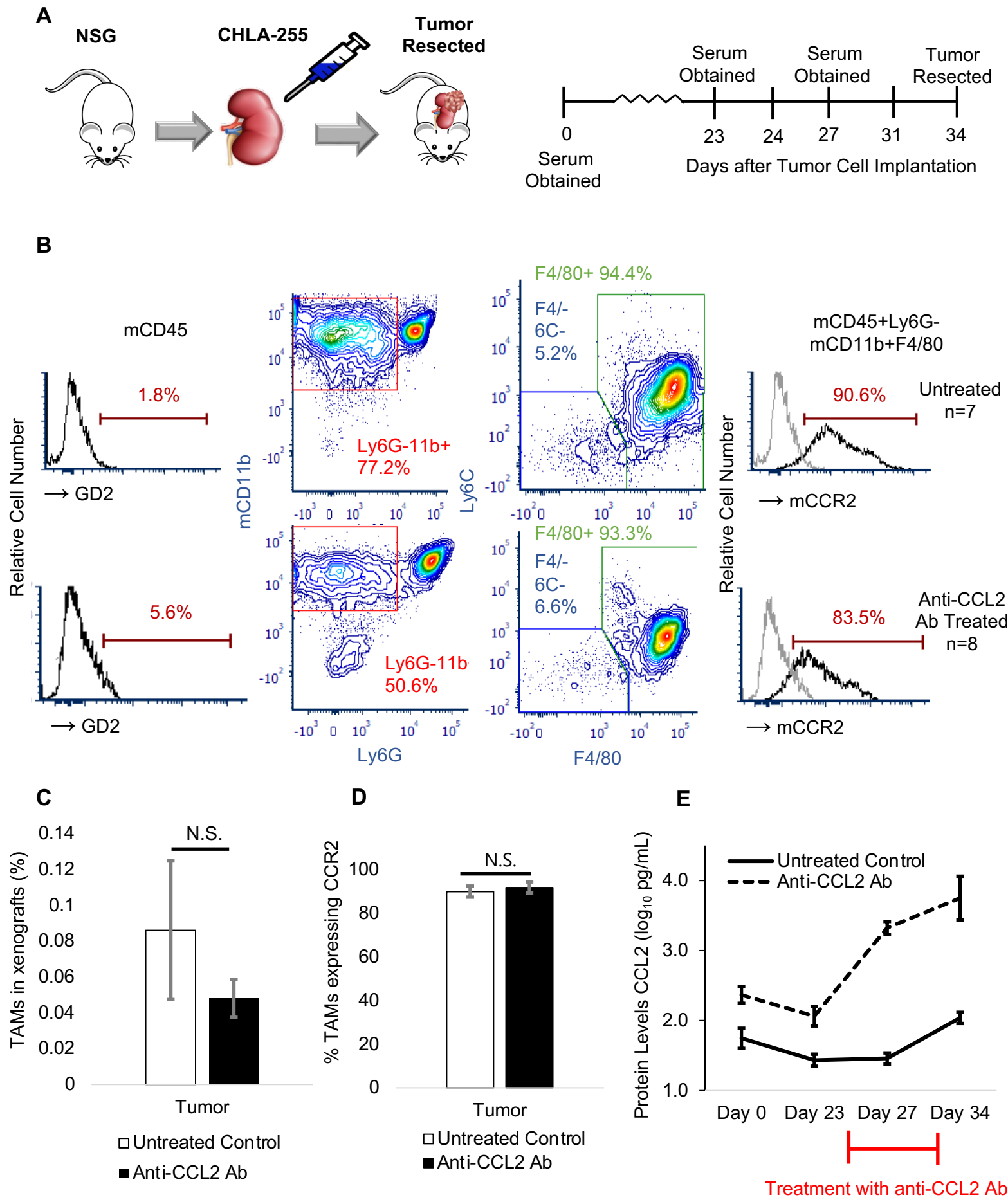

Supplement: Supplementary file 4 — Supplementary Figure S4. [file 41598_2023_46968_MOESM4_ESM.pdf]

Supplementary Figure S5

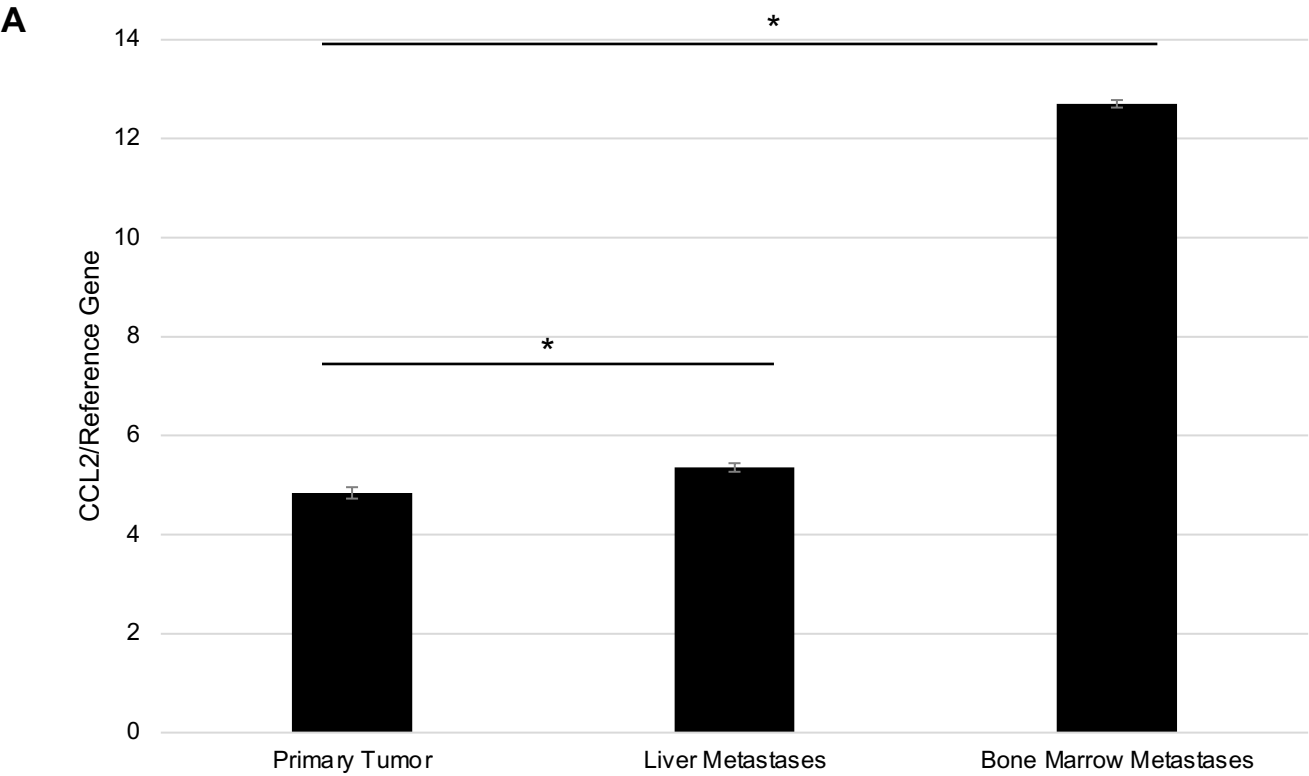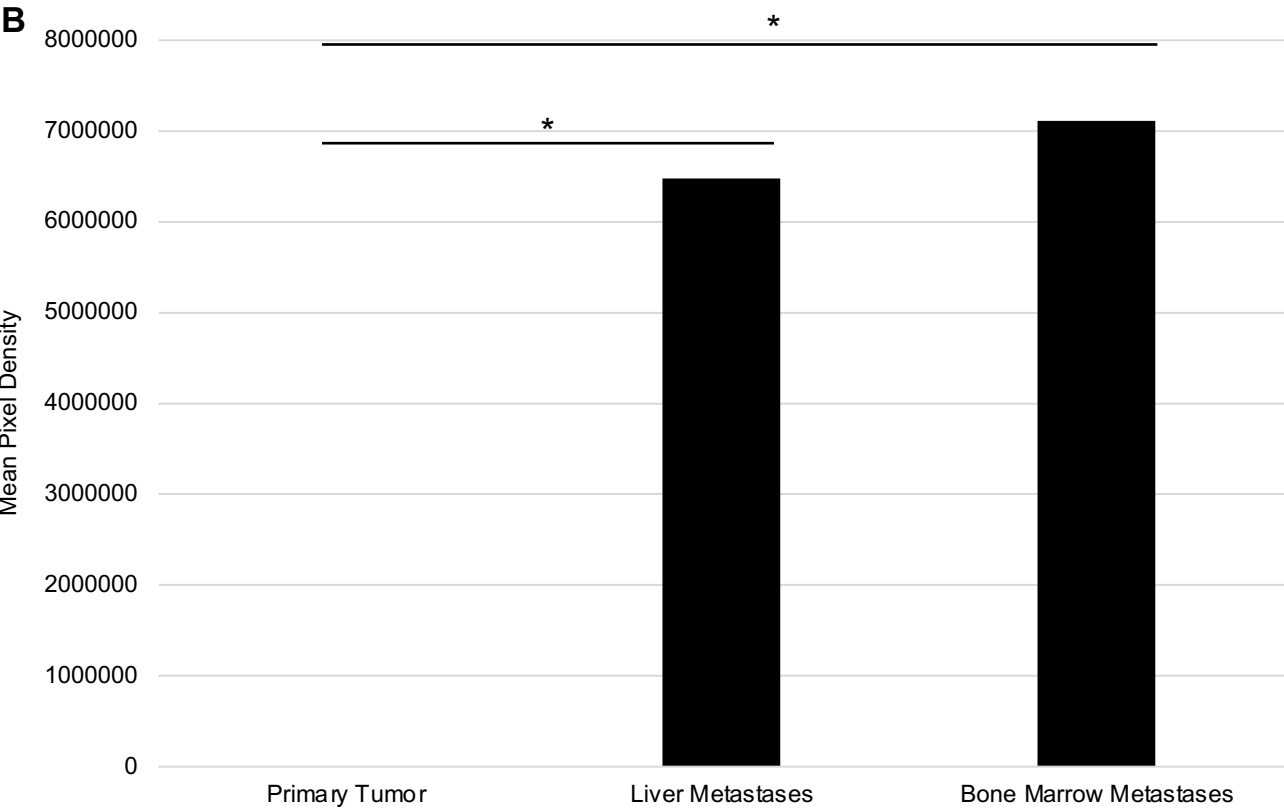

Supplement: Supplementary file 5 — Supplementary Figure S5. [file 41598_2023_46968_MOESM5_ESM.pdf]

Supplementary Figure S6

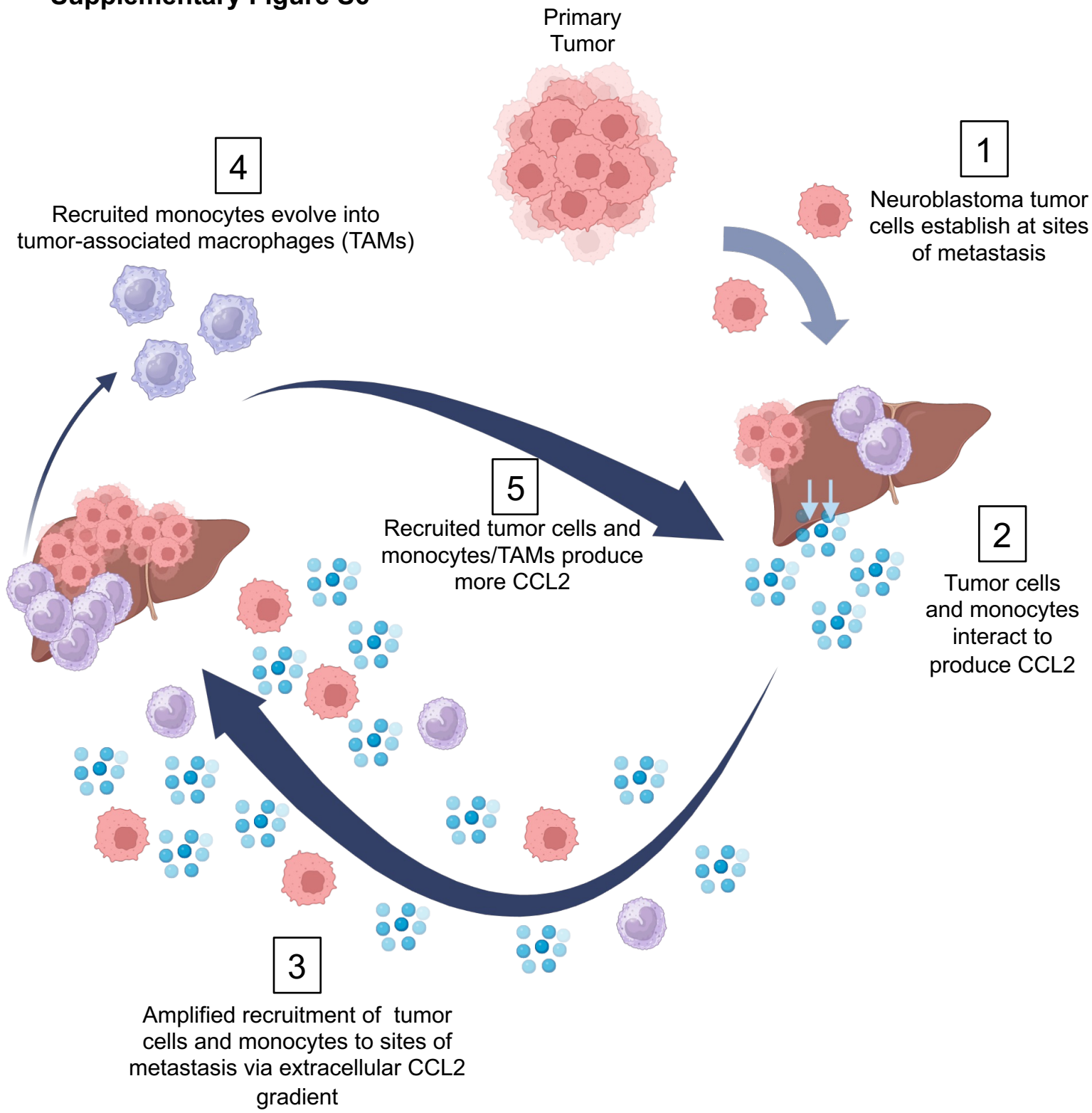

Supplement: Supplementary file 6 — Supplementary Figure S6. [file 41598_2023_46968_MOESM6_ESM.pdf]
