# Supplementary material for: Anti-CCL2 antibody combined with etoposide prolongs survival in a minimal residual disease mouse model of neuroblastoma
Source: Sci Rep. 2023 Nov 14;13:19915. doi: 10.1038/s41598-023-46968-2 (PMC10645976; doi:10.1038/s41598-023-46968-2)
Supplement: Supplementary file 7 — Supplementary Information 7. [file 41598_2023_46968_MOESM7_ESM.docx]

­­**Anti-CCL2 antibody combined with etoposide prolongs survival in a minimal residual disease mouse model of neuroblastoma**

Danny Lascano^+1^, Michael J. Zobel^+1^, William G. Lee^1^, Stephanie Y. Chen^1^, Abigail Zamora^1^, Grace E. Asuelime^1^, So Yung Choi^6^, Antonios Chronopoulos^2^, Shahab Asgharzadeh^2,3^, Araz Marachelian^2,3^, Jinseok Park^2^, Michael A. Sheard^4^, and Eugene S. Kim*^1,2,3,5^

1. Division of Pediatric Surgery, Children’s Hospital Los Angeles, Los Angeles, California, USA
2. Division of Hematology, Oncology and Blood and Marrow Transplantation, Keck School of Medicine of the University of Southern California, Los Angeles, California, USA
3. Department of Pediatrics, Keck School of Medicine of the University of Southern California, Los Angeles, California, USA
4. The Saban Research Institute, Children’s Hospital Los Angeles, Los Angeles, California, USA
5. Department of Surgery, Keck School of Medicine, University of Southern California, Los Angeles, California, USA
6. Biostatistics and Bioinformatics Research Center, Cedars-Sinai Medical Center, Los Angeles, California, USA

^+^These two authors contributed equally

***Corresponding author:**

Eugene S. Kim, MD, FACS, FAAP

Professor of Surgery and Pediatrics

Division of Pediatric Surgery

Division of Hematology and Oncology

Director, Division of Pediatric Surgery

Vice Chair for Pediatric Surgery, Department of Surgery

Cedars-Sinai Medical Center

116 N. Robertson Blvd, Suite PACT 700

Los Angeles, CA 90048

Phone: 310-423-6325

Fax: 310-248-8594

Email: eugene.kimx@cshs.org

**SUPPLEMENTARY METHODS**

**CCR2 expression studies**

Flow cytometry was performed to determine CCR2 expression in neuroblastoma (NB) cells. Four human NB cell lines (SH-SY5Y, CHLA-255-Fluc, NGP, SMS-KCNR) and one patient derived xenograft (COG-N-415X) were thawed from liquid nitrogen and re-suspended in culture media containing 10% fetal bovine serum (FBS). The cells were spun at 250 x g for 5 minutes at room temperature (RT) and re-suspended in cold PBS containing 0.5% Cytiva HyClone^TM^ bovine serum albumin (BSA) (Cytiva, Marlborough, MA; Catalog no. SH3057402) and 1 mM ethylenediaminetetraacetic acid (EDTA) (Sigma-Aldrich, Inc., St. Louis, MO; Catalog no. 324504) (FACS buffer). After counting, the cells underwent Fc-receptor blockade using Human TruStain FxC^TM^ (Fc Receptor Blocking Solution) (Biolegend ®, San Diego, CA; Catalog no. 422302) for 10 minutes in the dark and at room temperature (RT). The cells were then distributed into four separate flow cytometry tubes with one unstained control and the experimental samples in triplicates. The experiment samples were stained with mouse PerCP-Cy5.5 anti-human CD192 (CCR2) antibody (Biolegend ®, San Diego, CA; Catalog no. 335303) for 40 minutes in the dark at 4°C. The cells were subsequently washed in FACS buffer and stained with 4’,6-Diamidino-2-phenylindole (DAPI) (Sigma-Aldrich, St. Louis, MO; Catalog no. D9542) (0.1 ng/mL). Cells were passed through a 40 µm filtered cap tube and kept on ice prior to flow cytometry using a BD Biosciences LSR II (BD Biosciences, San Jose, CA). Flow cytometry data analysis was performed with BD FACSDiva^TM^ software (BD Biosciences, San Jose, CA).

**CCL2 expression in primary versus recurrent tumor tissue**

Primary tumors and recurrent tumors at sites of metastasis (liver, bone marrow) were obtained from neuroblastoma xenografts (CHLA-255 cell line) established according to the previously described murine model of minimal residual disease. Single-cell suspensions were created with neuroblastoma cell confirmation via flow cytometry as described previously. Fluorescence-based quantitative PCR was carried out on single-cell suspensions using the PowerUp^TM^ SYBR^TM^ Green Master Mix (Applied Biosystems, Invitrogen, Waltham, MA, USA) per manufacturer guidelines. Primers were designed using Primer Express 2.0 (Applied Biosystems, Invitrogen, Waltham, MA, USA) per software guidelines. Primer sequences used for quantitative PCR were: CCL2 forward (5’-AAGATCTCAGTGCAGAGGCTC-3’), CCL2 reverse (5’-TTGCTTGTCCAGGTGGTCCAT-3’), β-Actin forward (5′-GACAGGATGCAGAAGGAGATTACT-3′), β-Actin reverse (5′-TGATCCACATCTGCTGGAAGGT-3′). Absolute quantification was performed and results were expressed relative to the reference internal control gene, β-Actin. In addition, CCL2 protein expression was evaluated using the Proteome Profiler Human Angiogenesis Array kit (R&D Systems, Minneapolis, MN, USA) to analyze supernatant collected from primary tumor and recurrent tumor (liver, bone marrow) cell suspensions, per manufacturer guidelines.

**SUPPLEMENTARY TABLES**

**Supplementary Table 1**. Antibodies used in this study.

| Specificity | Fluorochrome | Cells Stained | Isotype | Clone | Vendor | Catalog # |
| --- | --- | --- | --- | --- | --- | --- |
| Anti-human CD56 | BUV395 | NEUROBLASTOMA cell line and tumor | Mouse BALB/c IgG2b, κ | NCAM16.2 | BD Biosciences | 563554 |
| Anti-mouse CCR2 | BV421 | Tumor | Rat IgG2b, κ | 475301 | BD Biosciences | 747963 |
| Anti-human GD2 | BV605 | NEUROBLASTOMA cell line and tumor | Mouse BALB/c IgG2a α | 14.G2a | BD Biosciences | 744071 |
| Anti-mouse Ly6C | AF488 | Tumor | Rat IgG2c, κ | HK1.4 | Biolegend | 128022 |
| Anti-mouse CD11b | PE | Tumor | Rat DA/HA IgG2b, κ | M1/70 | BD Biosciences | 557397 |
| Anti-mouse CD45 | PE-Cy7 | Tumor | Rat, LOU/M IgG2b, κ | 30-F11 | BD Biosciences | 552848 |
| Anti-F4/80 | APC | Tumor | Rat IgG2a, κ | BM8 | Biolegend | 123116 |
| Anti -Ly6G | AF700 | Tumor | Rat, Lewis IgG2a, κ | 1A8 | BD Biosciences | 561236 |

**Supplementary Table 2.** Linear mixed-model results for the log-transformed tumor flux in the mice implanted with NGP-Fluc neuroblastoma tumor cells across the four treatment groups (untreated control, anti-CCL2 Ab alone, etoposide alone, combination anti-CCL2 Ab and etoposide) up to day 23. Results demonstrate a similar increase in tumor flux (i.e. tumor burden) at day 16 and day 23 in the mice treated with anti-CCL2 Ab alone and etoposide alone, but a significant lack of increase in tumor flux at these timepoints in the mice treated with combination therapy (anti-CCL2 Ab and etoposide).

|  | Estimate | 95% CI | p-value |
| --- | --- | --- | --- |
| Intercept | 13.78 | 12.79 – 14.78 | <0.001 |
| Measurement Day (Ref: Day 9) |  |  |  |
| Day 16 | 1.35 | 0.63 – 2.07 | <0.001 |
| Day 23 | 2.20 | 1.49 – 2.92 | <0.001 |
| Group (Ref: Untreated Control) |  |  |  |
| Anti-CCL2 Ab | 0.19 | -1.22 – 1.60 | 0.788 |
| Etoposide | 0.08 | -1.33 – 1.49 | 0.911 |
| Anti-CCL2 Ab + Etoposide | 0.13 | -1.28 – 1.54 | 0.857 |
| Interaction b/w Day x Group |  |  |  |
| Day 16 x Anti-CCL2 Ab | -0.12 | -1.14 – 0.89 | 0.814 |
| Day 23 x Anti-CCL2 Ab | -0.19 | -1.21 – 0.82 | 0.710 |
| Day 16 x Etoposide | -0.64 | -1.66 – 0.37 | 0.212 |
| Day 23 x Etoposide | -0.13 | -1.15 – 0.89 | 0.800 |
| Day 16 x Anti-CCL2 Ab + Etoposide | -1.36 | -2.38 – -0.35 | 0.009 |
| Day 23 x Anti-CCL2 Ab + Etoposide | -1.58 | -2.60 – -0.57 | 0.003 |

**SUPPLEMENTARY FIGURE LEGENDS**

**Supplementary Figure S1.** Kaplan-Meier survival plots for event-free survival and overall survival in neuroblastoma tumor samples stratified by mean log2-transformed CCL2 RNA expression level. Analysis of published dataset from Cangelosi et al. (Cangelosi, 2020) **(S1A, S1B)** and unpublished dataset used by Asgharzadeh et al. (Asgharzadeh, 2005) **(S1C, S1D)** demonstrates no significant difference in event-free survival and overall survival based on CCL2 RNA expression level. ***(Please print in color)***

**Supplementary Figure S2.** CCR2 (CD192), the primary receptor for CCL2, is expressed in five different human neuroblastoma cell lines (CHLA-255, SMS-KCNR, SH-SY5Y, NGP, PDX (COG-N-415X)). Flow cytometry assays were performed in a minimum of replicates of three.

**Supplementary Figure S3.** SK-N-SH neuroblastoma cells migrate to increasing concentration gradients of CCL2, which is abrogated by anti-CCL2 antibody. Utilizing time-lapse live cell microscopy, measured displacement of SK-N-SH neuroblastoma cells towards varying CCL2 gradients was analyzed over time. Under conditions of no CCL2 (i) and CCL2 without a gradient (ii), there is no visualized neuroblastoma cell migration. Under the conditions of and increasing concentration gradient of CCL2 (iii), there is significant migration of neuroblastoma cells, which is abrogated by anti-CCL2 antibody (iv). Live cell microscopy data was performed in duplicate with over 100 cells measured per experiment. Linear regression analysis was performed. ***(Please print in color)***

**Supplementary Figure S4**. Tumor associated macrophages (TAMs) in neuroblastoma xenografts treated with anti-CCL2 antibody. (**A)** Schematic of experiment. Mice were intra-renally injected with 1 x 10^6^ CHLA-255 cells and xenografts were allowed to establish. Following sacrifice at day 37, tumors were micro-dissected and processed into single-cell suspension for flow cytometry to detect the presence of TAMs. Murine serum was obtained prior, during, and after treatment to measure CCL2 protein levels. Created with BioRender.com. (**B)** Representative image of flow cytometry showing TAM receptor expression in dissected tumor with untreated NSG mice demonstrating more TAMs than treated mice. (**C)** Pooled average of TAMs from treated and untreated neuroblastoma tumors. Decreased TAMs were found in treated mice compared to untreated mice. (**D)** CCR2 expression among TAMs from treated and untreated mice tumors were equivalent. (**E)** Murine plasma levels of CCL2 were markedly elevated in treated mice compared to untreated mice. The red line represents the period of treatment. Student’s t-test and linear regression was performed. Error bars representing mean + SEM; significance defined as p<0.05. ***(Please print in color)***

**Supplementary Figure S5**. **(A)** Fluorescence-based quantitative real-time PCR measuring CCL2 gene expression and **(B)** targeted proteomics assay measuring CCL2 protein expression in primary tumors and recurrent tumors (liver metastases, bone marrow metastases) from untreated neuroblastoma xenografts (CHLA-255 cell line). Increased gene and protein expression of CCL2 was found in the recurrent tumors compared to primary tumors. For the quantitative real-time PCR assay, absolute quantification was performed and results are expressed relative to the internal control gene, β-Actin. Student’s t-test was used to compare the CCL2 expression data between primary tumors and recurrent tumors (liver metastases, bone marrow metastases). Bar graphs and error bars represent mean ± SD; *p<0.01.

**Supplementary Figure S6.** Proposed mechanism of neuroblastoma tumor cell and monocyte recruitment to sites of metastasis via extracellular CCL2 secretion and gradient-dependent migration. Created with BioRender.com. ***(Please print in color)***
